# *Bartonella clarridgeiae* infection in a patient with aortic root abscess and endocarditis

**DOI:** 10.1099/acmi.0.000064

**Published:** 2019-10-10

**Authors:** Julie M. J. Logan, Jessica L. Hall, Vicki J. Chalker, Brian O’Connell, Richard J. Birtles

**Affiliations:** ^1^​ Bacterial Reference Department, Public Health England, London NW9 5EQ, UK; ^2^​ School of Science, Engineering and Environment, University of Salford, Manchester M5 4WT, UK; ^3^​ Microbiology Department, St James’s Hospital, Dublin 8, Ireland

**Keywords:** Endocarditis, zoonosis, *Bartonella*

## Abstract

**Introduction.:**

*
Bartonella* species are increasingly recognized as agents of culture-negative endocarditis. However, to date, almost all human cases have been associated with two members of the genus, *Bartonella henselae* and *Bartonella quintana. B. henselae* infections are zoonotic, with domestic cats serving as reservoir hosts for the pathogen. *Bartonella clarridgeiae* also exploits cats as reservoir hosts, but its zoonotic potential is far less established.

**Case presentation.:**

A 34-year-old male presented with palpitations after a history of aortic incompetence. During surgery for an aortic valve replacement, two vegetations were found on the aortic valve. PCR analysis of the vegetation demonstrated the presence of *Bartonella* species and so the patient was treated post-operatively with ceftriaxone and doxycycline, making a good recovery. Further PCR-based analysis of the patient’s aortic vegetation confirmed the presence of *B. clarridgeiae *.

**Conclusion.:**

This report expands the number of *Bartonella* species associated with endocarditis and provides clear evidence that *B. clarridgeiae* should be considered a zoonotic pathogen.

## Introduction

Members of the genus *Bartonella* are characterized as haemotrophic intra-erythrocytic bacterial parasites of a wide range of mammals and several members of the genus are established or emerging zoonotic pathogens. Although cat scratch disease is the most common zoonotic manifestation, a wide spectrum of other syndromes has also been encountered [1]. *Bartonella* species, in particular *Bartonella henselae* and *Bartonella quintana *, are now recognized as the second most common cause of blood culture-negative endocarditis [2].

*Bartonella clarridgeiae* has only rarely been implicated as a human pathogen; one study, based on serological diagnostics, reports its involvement in cat scratch disease and a chest wall abscess [3], whereas another reported its isolation from an asymptomatic human blood donor [1]. *B. clarridgeiae* is also recognized as a pathogen of veterinary importance, being associated with disease in dogs (endocarditis and hepatic disease) and cats (blindness and neuritis) [4, 5]. In this report we describe the detection of *B. clarridgeiae* DNA in a 34-year-old male with aortic root abscess and endocarditis. The species was identified based on comparative sequence analysis of PCR-amplified fragments of genes encoding 16S rRNA gene and citrate synthase.

## Case report

A 34-year-old male from Dublin, Ireland, presented with acute onset of palpitations with a 4-month history of malaise, night sweats and arthralgia. He was known to have aortic incompetence and had been regularly reviewed by a cardiology service. He had returned from worldwide travel approximately 1 year prior to admission and had an office-based job. He had no pets or unusual exposures from hobbies. The main finding on examination was of tachycardia; there were no stigmata of infective endocarditis. He had no fever on admission, but a transthoracic echocardiogram revealed a probable vegetation on a unicuspid aortic valve, which was confirmed by transoesophageal echocardiogram. Three sets of blood cultures were obtained prior to the initiation of empirical antimicrobial therapy, but were subsequently sterile.

Initial treatment consisted of flucloxacillin (2 g 4 h^−1^), benzylpenicillin (2.4 g 4 h^−1^) and gentamicin (1 mg kg^−^^1^ three times per day). However, when the blood cultures were confirmed as negative, the antimicrobials were changed to ceftriaxone (2 g daily), vancomycin (125 mg 6 h^−1^) and gentamicin (as above).

Laboratory investigations on admission revealed a neutrophil leucocytosis with elevated inflammatory markers. Serology for *B. henselae, B. quintana, Coxiella burnetii* and *Brucella* species were performed and were negative.

An aortic valve replacement was performed. Operative findings confirmed two vegetations on a unicuspid aortic valve with two abscess cavities. Microbiological investigations revealed no organisms on Gram stain of the infected valve and no growth following 10 days of incubation.

A specimen of aortic valve was submitted to the Bacterial Identification Section, Antimicrobial Resistance and Healthcare Associated Infections Unit, Public Health England for 16S rRNA gene sequencing, as described previously [6]. Briefly, DNA was extracted using the DNEasy Blood and Tissue kit (Qiagen Ltd, UK) and then incorporated as template in a real-time PCR. The reaction mix comprised 10 µl TaKaRa SYBR Premix Ex *Taq* (TaKaRa BioEurope, France), 0.4 µM each of forward primer CLSI (TTGGAGAGTTTGATCMTGGCTC) and reverse primer Bosshard (GTATTACCGCTGCTG), 4.4 µl water and 2 µl of DNA extract. Reaction mixes were exposed to a thermal cycle comprising initial denaturation at 95 °C for 10 s followed by 40 cycles of 95 °C for 15 s, 70 °C for 10 s and 72 °C for 20 s. PCR-based delineation of *Bartonella* species was subsequently achieved by comparative sequence analysis of a semi-nested PCR-amplified *gltA* fragment, as previously described [7, 8], performed at the University of Salford. Briefly, the first-round reaction mix comprised 25 µl MyTaq Red (Bioline Ltd, UK), 2 µl each of 10 pmol µl^−1^ forward primer 443 f (GCTATGTCTGCATTCTATCA) and reverse primer 1137 r (AATGCAAAAAGAACAGTAAACA), 19 µl of water and 2 µl of the sample DNA. The second-round reaction mix comprised 25 µl MyTaq Red, 2 µl each of 10 pmol µl^−1^ of forward primer 781 f (GGGGACCAGCTCATGGTGG) and reverse primer 1137 r, 20 µl of water and 1 µl of first-round product. The thermal cycle employed for both rounds of the assay consisted of 35 cycles of 95 °C for 30 s, 55 °C for 30 s and 72 °C for 60 s, with a final extension stage of 72 °C for 5 min. In both laboratories, rigorous procedures to prevent cross-contamination were employed. Sanger sequencing of the 16S rRNA gene and *gltA* amplicons yielded complete (928 base pair and 338 base pair, respectively) and unambiguous DNA sequences. Analysis of these sequences using Basic Local Alignment Search Tool (blast) and local databases revealed that the 16S rRNA gene fragment (GenBank accession number MG593163) was 99–100 % identical to those of *B. clarridgeiae* and *B. rochalimae* (accession numbers NR036961 and DQ683196, respectively)*,* whereas the *gltA* fragment (GenBank accession number MG384320) was 100 % identical to that of the type strain of *B. clarridgeiae* (accession number BCU84386) and differed from those of other *Bartonella* species by at least 4 % [the highest sequence similarity was 96.0 % with the type strain of *Bartonella rochalimae* (accession number DQ683195)].

Following the molecular detection of a *Bartonella* infection, the patient received ceftriaxone (2 g daily) and doxycycline (100 mg twice a day) for 6 weeks post-operatively and recovered well.

## Discussion

This report describes the molecular documentation of *B. clarridgeiae* DNA in human heart valve tissue. We believe that this case represents the first report of human infection by *B. clarridgeiae* in Ireland and the first report anywhere implicating *B. clarridgeiae* in aortic root abscess and endocarditis. Furthermore, it is the first time definitive molecular methods have been used to implicate this species in human disease rather than just serology, the specificity of which has been questioned as significant cross-reactions have been observed among *Bartonella* species and other pathogens [9]. Serum from our patient did not cross-react with either *B. henselae* or *B. quintana* antigens, an observation in keeping with one previous *B. clarridgeiae* case report [3] but contrary to another [10]. This inconsistency may reflect variation in the antigens used [11] and/or variation in host response to *Bartonella* infections [12].

We can only find evidence of a single case of aortic root abscess associated with *Bartonella* infection in the literature; *B. henselae* infection in a patient with a prosthetic aortic valve presenting with nonspecific constitutional symptoms, a skin rash and, subsequently, acute renal failure [13]. Conversely, several hundred cases of *Bartonella* endocarditis have now been recognized [2]; however, of those for which a specific *Bartonella* species has been implicated, over 95 % were associated with either *B. henselae* or *B. quintana *, with only a handful of cases attributed to other species, *Bartonella alsatica* and *Bartonella vinsonii* [2]. *B. henselae* and *B. quintana* represent the two most medically important *Bartonella* species worldwide and it is likely that their relative importance reflects the relatively high rate of human exposure to these species. *B. quintana* exploits humans as its reservoir host and is transmitted by body lice (*Pediculus humanus*) infections, and so is frequently associated with disease in those groups of society in which louse infestation is more common, such as homeless people and refugees [7]. *B. henselae* exploits domestic cats as its reservoir host, a species that has frequent and intimate contact with a significant proportion of the human population [14]. Although *B. clarridgeiae* is thought to exploit the same reservoir host, a survey of Irish cats revealed a far lower prevalence of *B. clarridgeiae* infection (0.8 %) than *B. henselae* infection (3.4 %), and hence a markedly diminished zoonotic hazard [14].

Given the similarities in the ecologies of *B. henselae* and *B. clarridgeiae *, it is likely that the zoonoses they cause share the same risk factors. The age of our patient (34) was close to the mean age of *B. henselae* endocarditis patients (39) and, like 67 % of these patients, he was male [2]. As with other *Bartonella* species, human zoonotic infection is likely to be mediated by arthropod vectors, although direct transmission may also occur; the first report of zoonotic *B. clarridgeiae* infection concerned a veterinarian who developed cat scratch disease following a cat bite [10].

The increasing use of PCR-based methods on aortic tissue will no doubt increase the detection of infectious endocarditis with *Bartonella* and other species [7]. However, the sensitivity of such an approach has been shown to be influenced by specimen type and the specific assay used [2], with *Bartonella* specific assays performing markedly better than a broad-range bacterial PCR (akin to that initially used in this study).

This case report indicates that *B. clarridgeiae* should be considered in cases of endocarditis and detection of infection may be achieved using PCR and amplicon sequencing on DNA extracted from heart valve tissue.

## References

[1] Vieira-Damiani G, Diniz PP, Pitassi LHU, Sowy S, Scorpio DG (2015). *Bartonella clarridgeiae* bacteremia detected in an asymptomatic blood donor. J Clin Microbiol.

[2] Edouard S, Nabet C, Lepidi H, Fournier P-E, Raoult D (2015). *Bartonella*, a common cause of endocarditis: a report on 106 cases and review. J Clin Microbiol.

[3] Margileth AM, Baehren DF (1998). Chest-Wall abscess due to cat-scratch disease (CSD) in an adult with antibodies to *Bartonella clarridgeiae*: case report and review of the thoracopulmonary manifestations of CSD. Clin Infect Dis.

[4] Chomel BB, Mac Donald KA, Kasten RW, Chang CC, Wey AC (2001). Aortic valve endocarditis in a dog due to *Bartonella clarridgeiae*. J Clin Microbiol.

[5] Gillespie TN, Washabau RJ, Goldschmidt MH, Cullen JM, Rogala AR (2003). Detection of *Bartonella henselae* and *Bartonella clarridgeiae* DNA in hepatic specimens from two dogs with hepatic disease. J Am Vet Med Assoc.

[6] Edwards KJ, Logan JMJ, Langham S, Swift C, Gharbia SE (2012). Utility of real-time amplification of selected 16S rRNA gene sequences as a tool for detection and identification of microbial signatures directly from clinical samples. J Med Microbiol.

[7] Chaloner GL, Harrison TG, Birtles RJ (2013). *Bartonella* species as a cause of infective endocarditis in the UK. Epidemiol Infect.

[8] Birtles RJ, Raoult D (1996). Comparison of partial citrate synthase gene (*gltA*) sequences for phylogenetic analysis of *Bartonella* species. Int J Syst Bacteriol.

[9] La Scola B, Raoult D (1996). Serological cross-reactions between *Bartonella quintana, Bartonella henselae,* and *Coxiella burnetii*. J Clin Microbiol.

[10] Kordick DL, Hilyard EJ, Hadfield TL, Wilson KH, Steigerwalt AG (1997). *Bartonella clarridgeiae*, a newly recognized zoonotic pathogen causing inoculation papules, fever, and lymphadenopathy (cat scratch disease). J Clin Microbiol.

[11] Maurin M, Rolain JM, Raoult D (2002). Comparison of in-house and commercial slides for detection by immunofluorescence of immunoglobulins G and M against *Bartonella henselae* and *Bartonella quintana*. Clin Vaccine Immunol.

[12] Yanagihara M, Tsuneoka H, Tanimoto A, Otsuyama K-I, Nishikawa J (2018). *Bartonella henselae* DNA in seronegative patients with cat-scratch disease. Emerg Infect Dis.

[13] Idrees J, Albacker TB, Gordon SM, Shin J, Menon V (2011). *Bartonella* infective endocarditis of a prosthetic aortic valve with a subvalvular abscess. J Card Surg.

[14] Juvet F, Lappin MR, Brennan S, Mooney CT (2010). Prevalence of selected infectious agents in cats in Ireland. J Feline Med Surg.

